# The brown and brite adipocyte marker Cox7a1 is not required for non-shivering thermogenesis in mice

**DOI:** 10.1038/srep17704

**Published:** 2015-12-04

**Authors:** Stefanie F. Maurer, Tobias Fromme, Lawrence I. Grossman, Maik Hüttemann, Martin Klingenspor

**Affiliations:** 1 Chair of Molecular Nutritional Medicine, Technische Universität München, Else Kröner-Fresenius Center for Nutritional Medicine & ZIEL-Institute for Food and Health, 85350 Freising-Weihenstephan, Germany; 2 Center for Molecular Medicine and Genetics, Wayne State University School of Medicine, Detroit, MI 48201, USA

## Abstract

The cytochrome *c* oxidase subunit isoform Cox7a1 is highly abundant in skeletal muscle and heart and influences enzyme activity in these tissues characterised by high oxidative capacity. We identified Cox7a1, well-known as brown adipocyte marker gene, as a cold-responsive protein of brown adipose tissue. We hypothesised a mechanistic relationship between cytochrome *c* oxidase activity and Cox7a1 protein levels affecting the oxidative capacity of brown adipose tissue and thus non-shivering thermogenesis. We subjected wildtype and Cox7a1 knockout mice to different temperature regimens and tested characteristics of brown adipose tissue activation. Cytochrome *c* oxidase activity, uncoupling protein 1 expression and maximal norepinephrine-induced heat production were gradually increased during cold-acclimation, but unaffected by Cox7a1 knockout. Moreover, the abundance of uncoupling protein 1 competent brite cells in white adipose tissue was not influenced by presence or absence of Cox7a1. Skin temperature in the interscapular region of neonates was lower in uncoupling protein 1 knockout pups employed as a positive control, but not in Cox7a1 knockout pups. Body mass gain and glucose tolerance did not differ between wildtype and Cox7a1 knockout mice fed with high fat or control diet. We conclude that brown adipose tissue function in mice does not require the presence of Cox7a1.

Brown adipose tissue (BAT) provides heat production as an adaptive mechanism for the maintenance of normothermia in response to low environmental temperatures. Since this non-shivering thermogenesis (NST) results in the dissipation of stored energy, the function of BAT is entirely opposite to the energy-saving properties of white adipose tissue (WAT). Besides the brown and white adipocytes of their respective, classical depots, a third type of adipocyte was found to occur in the adipose organ of rodents^1^. Due to its morphological appearance and distinct anatomical localization in WAT depots, this novel cell type is termed brite cell (brown-in-white)^2^. The abundance of these cells is triggered by cold-exposure^1^ and adrenergic stimulation^3^. Although brown and brite adipocytes are not derived from the same developmental lineage^4^, both cell types are first and foremost characterised by a multilocular appearance, high mitochondrial abundance and the expression of mitochondrial uncoupling protein 1 (Ucp1). The latter is considered to be the key thermogenic factor mediating NST in BAT depots. Thus, brite cells are also equipped with the appropriate molecular requirements to provide increased energy expenditure via mitochondrial uncoupling when adequately stimulated^5^,^6^.

The number of brown and brite adipocytes in response to a certain treatment can be approximated by the determination of marker gene expression via qRT-PCR. For that purpose, a multitude of candidates is available^7^,^8^ and the list of these brown-selective markers is constantly growing. To extend the relevance of these molecules from only a marker to a functionally important component, the use of genetically modified mice represents an appropriate strategy. This has, however, by far not been investigated for the majority of candidates that come into question. One interesting candidate is Cox7a1. Although widely used as a brown-selective marker gene, the functional role of this molecule in BAT and WAT has so far not been elucidated. Data from our previous study^9^ indicated that Cox7a1 is a highly cold-responsive protein of BAT. This not only confirms its classification as a brown-selective target on a higher level, but also hints towards a central role of Cox7a1 for brown adipocyte associated metabolism. Since BAT is the major site for adaptive heat production in mammals exposed to low environmental temperatures, we hypothesised that cold-induced upregulation of Cox7a1 in BAT might contribute to that process via modification of cytochrome *c* oxidase (COX) activity.

COX is a complex of the mitochondrial respiratory chain and uses cytochrome *c* as substrate to catalyse the reduction of molecular oxygen to water, which is the terminal and rate-limiting step of mitochondrial respiration in intact cells^10^,^11^. COX is a dimeric enzyme^12^ consisting of 13 individual subunits per monomer^13^. Interestingly, at least five subunits of the COX-monomer are expressed as different isoforms and the assembly of COX from a different set of isoforms constitutes one important level of activity regulation^14^,^15^,^16^,^17^,^18^. One of these is subunit 7a (Cox7a), which has two isoforms encoded by separate nuclear genes: Cox7a2, with ubiquitous expression, and Cox7a1, which is most abundantly expressed in heart and skeletal muscle, where it accounts for 60% (heart) and 50% (muscle) of total Cox7a abundance^19^. The particular molecular function of this subunit and its isoforms in the holoenzyme remains elusive. Cox7a has been described as a late-stage assembly subunit that integrates peripherally into the pre-existing complex and might be involved in the maturation of the final holoenzyme^20^.

Similar to heart and skeletal muscle, BAT represents a tissue of exceptional oxidative capacity. Cold-exposure increases this capacity and at the same time strongly affects Cox7a1 protein abundance^9^. Due to its high abundance in heart and skeletal muscle, ablation of Cox7a1 in mice results in reduced COX-activity in these two tissue types along with the development of cardiac myopathy and impaired skeletal muscle function^21^,^22^. These findings prompted us to look into BAT as a third tissue with very high Cox7a1 abundance and to potentially revise the view of Cox7a1 solely as a marker towards a molecule with functional relevance for brown adipocytes. To test this, we employed wildtype and Cox7a1 knockout mice.

## Results

### Respiratory chain complexes in brown adipose tissue (BAT) are remodelled in response to cold

**Figure 1:**
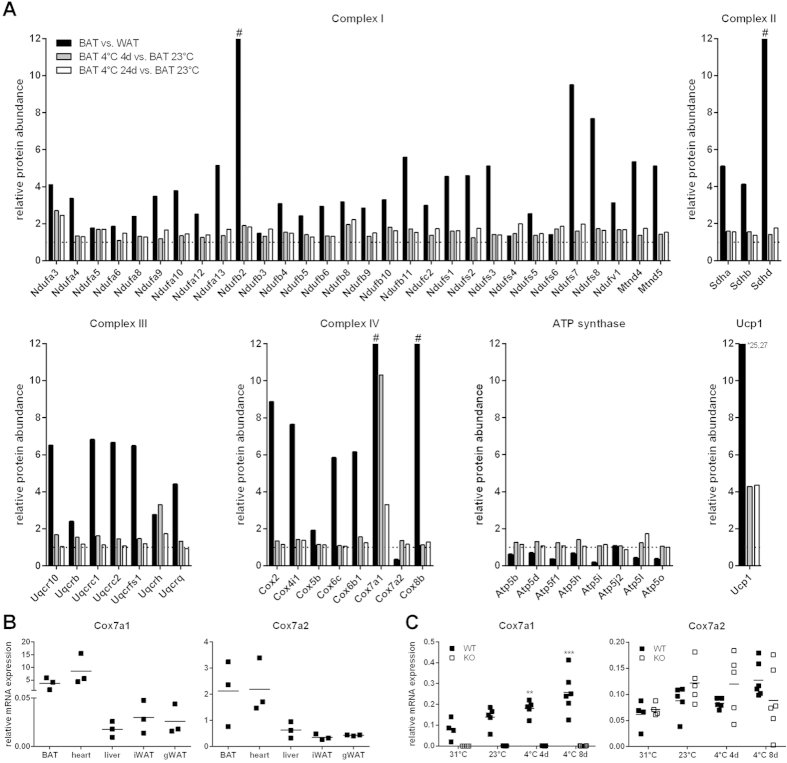
Cox7a1 is a cold-responsive protein of BAT and might contribute to NST capacity. (**A**) Data of our previous study^9^. Abundance of BAT and WAT mitochondrial proteins was determined by LC-MS/MS. Data are summarised for proteins that can be assigned to respiratory chain complexes. Depicted proteins were selected from datasets based on their identification in two measurements indicating relative protein expression in (1) BAT vs. WAT and (2) BAT of cold-exposed mice (4 °C for 4 or 24 days, respectively) vs. BAT of mice housed at room temperature (23 °C). # indicates BAT-unique isoforms not detected in WAT. Y-axis for Ucp1 was set to 12 and *indicates actual protein abundance in BAT vs. WAT. (**B**) Relative mRNA levels of Cox7a1 and Cox7a2 in BAT, heart, liver, subcutaneous inguinal WAT (iWAT), and visceral gonadal WAT (gWAT) of WT mice housed at 23 °C (n = 3). Data are normalised to general transcription factor 2b (Gtf2b) expression. (**C**) Relative Cox7a1 and Cox7a2 mRNA levels in BAT of WT and Cox7a1-KO mice either housed at thermoneutrality (31 °C) for 2 weeks, at 23 °C, or at 4 °C for 4 or 8 days (n = 4–6). Data are normalised to Gtf2b, Eef2, Tbp, and Hsp90ab1 expression. Gene expression was analysed using Two Way ANOVA and Holm-Sidak post-test. Asterisks (**p < 0.01; ***p < 0.001) indicate significant difference compared to WT at 31 °C.

BAT is rich in mitochondria and has a very high respiratory capacity as compared to other tissues. Indeed, in a previous study we quantified mitochondrial proteins in brown and white adipose tissue (WAT)^9^ and identified subunits of mitochondrial respiratory chain complexes to be BAT-enriched. We found a multitude of subunits as well as uncoupling protein 1 (Ucp1) to be expressed at a higher level in BAT compared to WAT, or to be exclusively expressed in BAT but not WAT (BAT unique; Fig. 1A). Furthermore, we described cold-induced changes in a total number of 264 mitochondrial proteins in BAT of mice upon acute (4 days) or chronic (24 days) cold-exposure (4 °C) compared to room temperature-acclimated controls. Among them were many subunits of respiratory chain complexes (Fig. 1A). Besides Ucp1 (>4 fold increased at 4 °C), we identified the BAT unique Complex IV subunit 7a isoform 1 (Cox7a1) as a highly cold-responsive protein (>10 fold and >3 fold increased after acute and chronic cold-exposure, respectively; Fig. 1A). In contrast, the expression of the alternative isoform, Cox7a2, is decreased in BAT compared to WAT and is far less cold-responsive in BAT than Cox7a1. This suggests that Cox7a1, but not Cox7a2, plays an essential role in BAT-derived thermogenesis. Cox7a1 is also termed heart-type isoform (Cox7ah), since it is most abundantly expressed in tissues with a high aerobic capacity such as heart and skeletal muscle. Indeed, Cox7a1 and Cox7a2 mRNA abundance in BAT was similar to the high transcript levels in heart as compared to liver and WAT depots (Fig. 1B), indicating the two isoforms to be of similar importance for cytochrome *c* oxidase (COX) activity in BAT and heart. In fact, Cox7a1 ablation reduces COX-activity in heart and skeletal muscle^21^,^22^.

These observations suggest that increased respiratory capacity of BAT in a cold environment may not only be caused by an overall increase in mitochondrial protein abundance, but also by an exchange of isoforms of Complex IV subunits. We hypothesised that Cox7a1 replaces Cox7a2 during cold-induced remodelling of BAT mitochondria, thereby increasing COX-activity and resulting in an increased capacity for non-shivering thermogenesis (NST). To test this hypothesis, we employed mice carrying a constitutive knockout of the Cox7a1 allele.

To validate our previous observations, we measured Cox7a1 and Cox7a2 transcript levels in BAT of wildtype (WT) and homozygous knockout (KO) mice either housed at thermoneutrality (31 °C for 2 weeks), at room temperature (23 °C), or at 4 °C (cold-exposed; Fig. 1C). Cox7a1 transcripts were not present at any condition in Cox7a1 ablated mice. In cold-exposed WT mice, Cox7a1, but not Cox7a2, mRNA expression was increased compared to mice housed at room temperature or thermoneutrality, supporting the results of our proteome study at the transcript level. At any temperature, Cox7a2 levels of KO mice were similar to WT mice, indicating that the KO of Cox7a1 is not compensated by alterations in the expression of the alternative isoform.

### Cold induced recruitment of respiratory capacity in BAT is independent of Cox7a1

**Figure 2:**
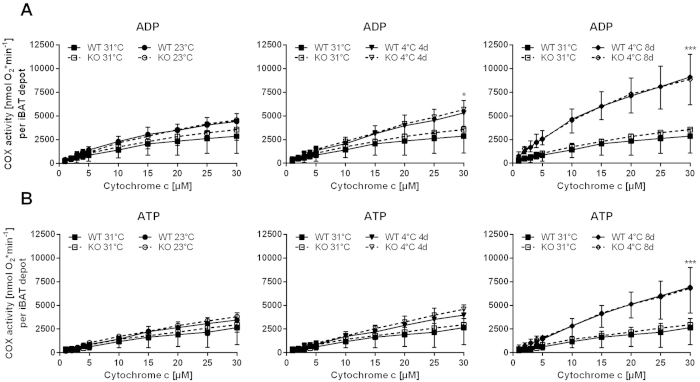
Cytochrome *c* oxidase (COX) activity in WT and Cox7a1-KO mice that were housed at 31 °C for 2 weeks, at 23 °C, or at 4 °C for 4 or 8 days (n = 4–6). Oxygen consumption was measured in iBAT homogenates in the presence of 5 mM ADP (**A**) or an ATP-regenerating system (**B**) at increasing concentrations of cytochrome *c* (1–30  μM). Oxygen consumption was statistically evaluated at the endpoint (30 μM) by Two Way ANOVA and Holm-Sidak post-test. Asterisks indicate significant effect of temperature (***p < 0.001).

Cox7a1 is a cold-responsive protein of BAT and serves as one of two alternative isoforms for COX subunit 7a. Ablation of Cox7a1 may thus modulate COX-activity in cold-exposed animals. To test this, we measured the activity of solubilised COX in BAT homogenates of mice housed at different temperatures. COX-activity was recorded at increasing concentrations of cytochrome *c* (1–30 μM). Oxygen consumption was recorded in the presence of ADP (Fig. 2A) or of ATP (Fig. 2B). The latter acts as an allosteric inhibitor^23^, thus leading to lower oxygen consumption compared to the presence of the allosteric activator ADP^24^. In both measurements, COX-activity was lowest in BAT from animals housed at thermoneutrality. Reduction of housing temperature to 23 °C and 4 °C resulted in a stepwise increase in COX-activity. The effect of cold exposure was duration-dependent, culminating in a large increase in COX-activity after 8 days at 4 °C. Surprisingly, neither absolute COX-activity of mice from any acclimation state, nor the magnitude of adaptive response, was in any way affected by Cox7a1 ablation.

**Figure 3:**
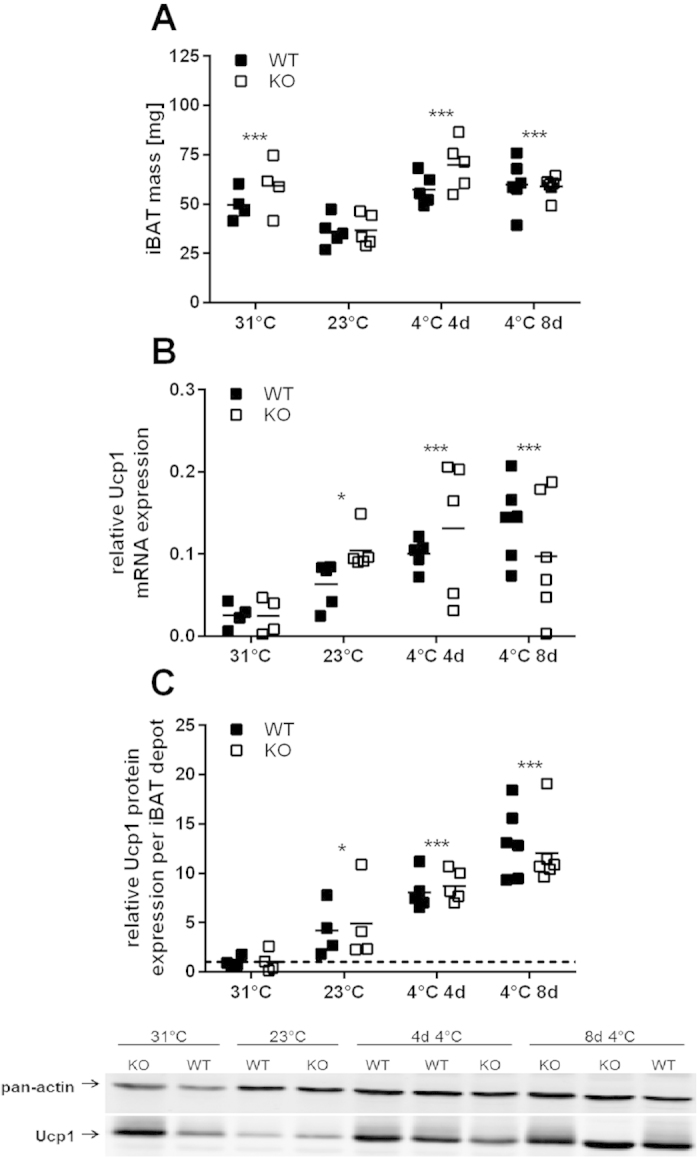
Characteristics of BAT recruitment in WT and Cox7a1-KO mice that were either housed at 31 °C for 2 weeks, at 23 °C, or at 4 °C for 4 or 8 days (n = 4–6). Data were analysed using Two Way ANOVA and Holm-Sidak post-test (*p < 0.05; ***p < 0.001). Significant genotype effects were not detected in the depicted datasets. (**A**) Dissected iBAT wet tissue mass. Asterisks indicate significant difference from 23 °C. (**B**) Relative Ucp1 mRNA levels in BAT. Data are normalised to Gtf2b, Eef2, Tbp, and Hsp90ab1 expression. Asterisks indicate significant difference from 31 °C. (**C**) Relative Ucp1 protein levels in total iBAT depots. Data are normalised to pan-actin expression and expressed as fold-change of expression levels observed in WT at 31 °C. Asterisks indicate significant difference from 31 °C. A full Western Blot image is provided in Supplementary Figure 1.

Cox7a1 protein is putatively involved in heat production in BAT and its ablation may affect characteristics of BAT recruitment other than COX-activity. We thus monitored temperature dependent changes in BAT mass and Ucp1 expression. Mass of the interscapular BAT depot (iBAT) was only marginally affected by the different temperature regimens. The lowest mass was observed in mice housed at room temperature and was comparable in mice housed at 31 °C and 4 °C (Fig. 3A). Tissue mass was not affected by genotype. Similar to COX-activity, Ucp1 transcript (Fig. 3B) and protein (Fig. 3C) expression were lowest in animals housed at thermoneutrality and stepwise increased in response to cold. Protein expression was increased more than 4-fold at 23 °C, indicating that conventional housing temperatures already represent a state of mild cold-exposure, recruiting thermogenesis. At 4 °C, Ucp1 protein levels were even more increased (>8 fold and >12-fold after 4 days and 8 days, respectively). Transcript and protein levels in KO mice were similar to WT in all conditions. Ablation of Cox7a1 does not affect Ucp1 expression in BAT.

**Figure 4:**
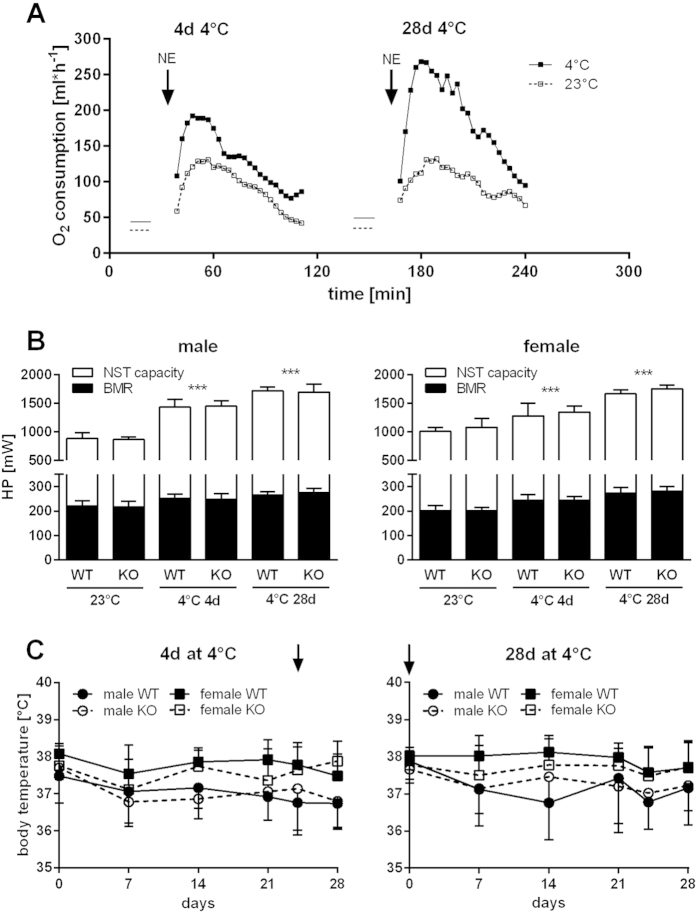
BAT-derived heat production in WT and Cox7a1-KO mice. Male and female mice of both genotypes were subjected to two consecutive norepinephrine (NE) tests performed at room temperature-acclimated state (23 °C, first test) and after acute (4 days) or chronic (4 weeks) cold-exposure (4 °C, second test; n = 5 in all groups). (**A**) Representative oxygen consumption traces from NE-tests exemplarily depicted for WT mice. Oxygen consumption of mice was recorded before (BMR, indicated as horizontal line) and after the injection of NE. (**B**) Basal (BMR) and NE-induced BAT-derived (NST capacity) heat production (HP). The latter was calculated as difference between maximal NE-stimulated HP (NE_max_) and BMR. Statistical analysis was conducted using Two Way ANOVA and Holm-Sidak post-test. Asterisks indicate significant differences between cold-exposure and room temperature. These differences were present in all three parameters tested (BMR, NE_max_ and NST capacity) with identical degree of significance (***p < 0.001). Significant differences between genotypes were not detected. (**C**) Rectal body temperature of WT and KO mice during the experiment. Arrows indicate the onset of cold-exposure in the respective groups. Statistical analysis was conducted separately for males and females of the two treatment groups. Differences in body temperature between WT and KO animals over time were tested by Two Way ANOVA and Holm-Sidak post-test.

Beyond direct effects on COX-activity or Ucp1 expression, Cox7a1 may contribute to cold-induced thermogenic capacity of BAT by other mechanisms. We therefore determined maximal norepinephrine (NE) stimulated heat production (HP) in WT and Cox7a1-KO mice. Each animal was injected twice with NE, thus assessing the capacity for NST in a room temperature-acclimated state and after acute (4 days) or chronic (28 days) exposure to 4 °C. Oxygen consumption of mice was recorded via indirect calorimetry and injection of NE resulted in a characteristic rapid increase of oxygen consumption, reaching a peak after 15–20 min, and referred to as maximal norepinephrine-stimulatable respiration (NE_max_). Both basal metabolic rate (BMR) and NE_max_ were increased after only 4 days at 4 °C and even more elevated after prolonged cold-exposure (Fig. 4A,B). The temperature dependency of HP at NE_max_ was well reflected in NST capacity, which was calculated as the difference in HP between NE_max_ and BMR (Fig. 4B). Due to reduced housing temperature and prolonged exposure time, NST capacity gradually increased. Correspondingly, rectal body temperature was well maintained and fairly constant in all groups throughout the experiment (Fig. 4C). Females tended to display a higher body temperature compared to males (38 °C vs. 37 °C), although NST capacity was comparable between both sexes at all temperature conditions tested. We could not, however, observe a difference between the genotypes at any condition, indicating BAT-derived adaptive heat production to be unaffected by Cox7a1 ablation.

### BAT activity visualised by thermal imaging is not affected by Cox7a1 ablation

Small mammals rely on BAT-mediated NST to maintain normothermia in a subthermoneutral environment. New-born mice represent an ideal model to visualise BAT activity by infrared thermal imaging since they lack protective fur. This not only increases dermal heat loss and thus HP but also overcomes technical impediments for the application of thermal imaging devices. As control, we employed Ucp1 knockout mice, known to exhibit defective BAT-derived thermogenesis^25^. Heterozygous breeding pairs were used to obtain offspring of all genotypes. Entire litters were subjected to thermal imaging within the first three days of life. Pups were placed face-down into multiwell plates and an individual series of pictures was taken from each litter. Interscapular skin surface temperature (iSST) was determined as measure for BAT activity and litter differences were standardised as outlined in the Methods section.

**Figure 5:**
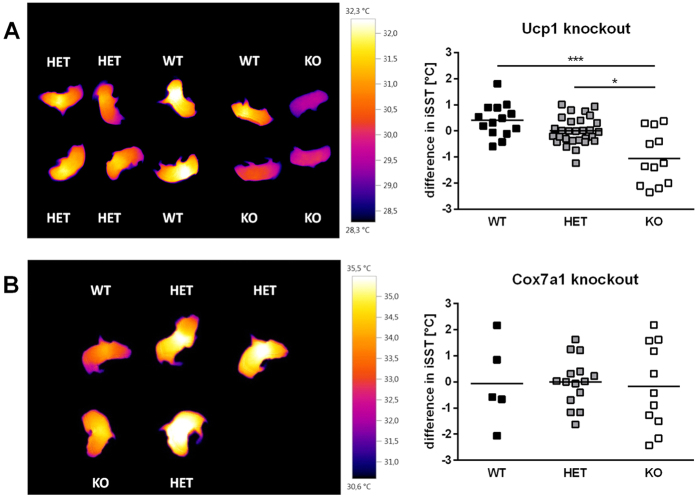
Thermal imaging of new-born pups from (A) Ucp1 (N = 7, n = 12–29) and (B) Cox7a1 (N = 5, n = 5–15) knockout breeding pairs. Pups were derived from heterozygous breeding and entire litters were subjected to imaging. Interscapular skin surface temperature (iSST) was used as measure for BAT activity. Data were analysed by One Way ANOVA on Ranks and Dunn’s multiple comparison test (*p < 0.05; ***p < 0.001).

In most pups, the area with the highest temperature was located in the interscapular region and around the neck. Among Ucp1 knockout mice we found WT and heterozygous (HET) mice to have comparable iSST. On average, homozygous Ucp1-KO mice displayed a more than 1 °C lower iSST compared to both WT and HET mice (Fig. 5A). Although maturation of BAT in altricial mammals such as mice occurs shortly after birth^26^,^27^,^28^, thus reducing heat production in earlier postnatal stages^29^, these results clearly indicate that infrared imaging of iSST in new-born mice is a suitable method to detect defective iBAT activity. Accordingly, we applied this technique to neonates obtained from heterozygous Cox7a1 breeding pairs (Fig. 5B). The analysis did not reveal differences in iSST between WT, HET, and KO animals, thus indicating that Cox7a1 ablation does not affect BAT-activity in new-born mice.

### The abundance of brite cells in WAT is affected by cold-exposure but not Cox7a1 ablation

**Figure 6:**
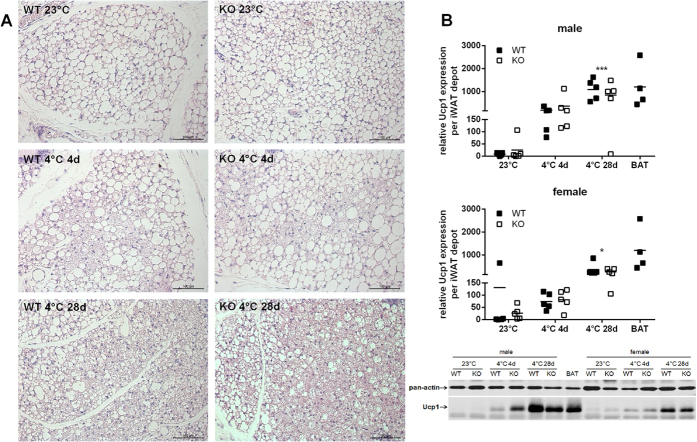
Characteristics of WAT browning in WT and Cox7a1-KO mice either housed at room temperature (23 °C) or at 4 °C for 4 or 28 days (n = 5). (**A**) HE-stained sections of inguinal WAT (iWAT). (**B**) Relative UCP1 protein expression iWAT. Data were normalised to pan-actin expression. Statistical analysis was conducted using Two Way ANOVA and Holm-Sidak post-test. Asterisks indicate significant effect of cold-exposure (*p < 0.05; ***p < 0.001) compared to 23 °C. Significant differences between genotypes were not detected. A full Western Blot image is provided in Supplementary Figure 2.

Beyond its function as a marker for brown adipocytes of BAT, Cox7a1 is a frequently used marker to substantiate the presence of brite adipocytes in WAT depots prone to browning. Accordingly, we asked whether ablation of Cox7a1 affects cold-induced brite cell-recruitment. On the morphological level, we observed a gradual increase in the abundance of multilocular cells in inguinal WAT (iWAT) after 4 days or 4 weeks at 4 °C compared to 23 °C (Fig. 6A). In line with this finding, Ucp1 protein expression in iWAT was considerably upregulated due to cold-exposure (Fig. 6B), reaching expression levels after 4 weeks at 4 °C comparable to what is observed in iBAT at 23 °C. Male mice tended to be more responsive compared to female mice. Both the abundance of multilocular cells and Ucp1 protein were, however, not influenced by genotype, indicating the recruitment of brite cells to be affected by cold-exposure but not Cox7a1 ablation.

### Ablation of Cox7a1 does not affect the metabolic response to high fat diet (HFD) feeding under thermoneutral conditions

BAT is hypothesised to be involved in the metabolic control of body mass in response to hypercaloric feeding^30^. Such regulation may be masked at subthermoneutral environment when BAT is active to provide HP for adaptive NST. Indeed, WT and Cox7a1-KO mice housed at room temperature on regular chow diet did not differ in body mass irrespective of gender (data not shown). This prompted us to investigate metabolic effects of HFD feeding in WT and Cox7a1-KO mice housed at 31 °C, thus eliminating the need for BAT-derived NST to uncover potential implications of putatively defective BAT-function in the development of diet-induced obesity (DIO).

**Figure 7:**
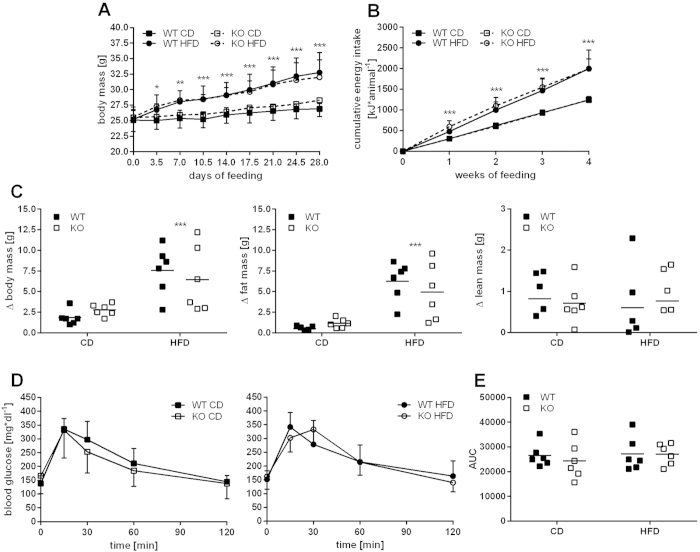
Feeding experiment with WT and Cox7a1-KO mice at 31 °C. Mice were housed in climate cabinets and fed for 4 weeks either with control (CD) or high fat (HFD) diet (n = 6). Datasets were analysed using Three Way ANOVA (A + B), Two Way ANOVA (C + E) and Two Way RM ANOVA (D) and Holm-Sidak post-test. Asterisks indicate significant effect of HFD-feeding (*p < 0.05; **p < 0.01; ***p < 0.001). Significant differences between genotypes were not detected in all depicted datasets. (**A**) Body mass development and (**B**) cumulative food intake throughout the experiment. (**C**) Difference in body, fat and lean mass between beginning and end of HFD feeding. (**D**) Blood glucose levels and (**E**) total area under the curve (AUC) measured during oral glucose tolerance test (oGTT) at the end of the experiment.

As expected, there was a difference in body mass development between control diet (CD) and HFD-fed mice, which was already observable 3–4 days after the start of HFD feeding (Fig. 7A). Accordingly, total body mass gain was higher in HFD-fed mice compared to CD-fed mice, but similar between WT and KO animals under both conditions (Fig. 7C). Body mass gain of WT and KO mice on HFD was largely attributable to an increased fat mass gain (Fig. 7C). In line with this, WT and KO animals on HFD had an increased energy intake compared to CD-fed mice during the entire 4 week feeding-period (Fig. 7B). Glucose tolerance was similar in WT and KO mice both under CD and HFD feeding (Fig. 7D). Furthermore, we observed similar glucose tolerance between CD- and HFD-fed mice in both genotypes (see area under the curve (AUC) in Fig. 7D). HFD-fed WT mice of the C57BL/6J strain are known to be prone to DIO and HFD-induced impairment of glucose tolerance when housed at room temperature^31^. Housing under thermoneutral conditions appears to abrogate this effect on glucose tolerance, but not on DIO development. Neither body mass development nor glucose tolerance, however, was influenced under HFD-feeding by the presence or absence of Cox7a1, indicating that the metabolic function of BAT is not altered due to Cox7a1 ablation.

## Discussion

The globally increasing prevalence of obesity constitutes the recruitment and activation of brown and brite adipocytes as novel targets in the development of counterregulatory treatment strategies. The rediscovery of functional brown adipose tissue (BAT) in humans^32^,^33^,^34^,^35^ has stimulated a boost of BAT-centred research during the last years^36^. Indeed, BAT activation in humans seems promising since it was recently associated with an increase in energy expenditure in response to cold-exposure^37^,^38^,^39^ or pharmacological treatment^40^. Accordingly, it is necessary to deepen our understanding of the indispensable components of the molecular machinery underlying the control of BAT activity. Cox7a1 seemed to be a promising target for three reasons:

1. As demonstrated (Fig. 1A), Cox7a1 protein is upregulated in BAT of mice exposed to a cold environment, indicating an association between Cox7a1 expression and adaptive BAT recruitment. A knockout mouse model was readily available to test this relationship.
2. Heart and skeletal muscle are affected by knockout of Cox7a1^21^,^22^ due to the high abundance of this isoform. BAT and heart have similar expression levels of Cox7a1 and Cox7a2 (Fig. 1B). This suggested the two isoforms to be of similar importance for cytochrome *c* oxidase (COX) activity in these tissues. Knockout of Cox7a1 might thus result in putative defects of BAT function due to altered COX-activity.
3. Cox7a1 is one of several marker genes that were consistently identified to be enriched in brown vs. white adipose tissue (WAT) as well as to be upregulated in WAT upon cold-stimulation, i.e., during browning^8^, thus serving as a transcriptional fingerprint for the identification of brown adipocytes in both tissues. Among these candidates, BAT associated phenotypes in mice have been reported for Ucp1^25^, Fabp3^41^, Cpt1b^42^, Dio2^43^, and Acot11^44^, extending the relevance of these proteins from a sheer marker to a functionally relevant component. The majority of candidates, including Cox7a1, have not been investigated so far.

Since Cox7a1 is a subunit isoform of cytochrome *c* oxidase (COX) of the respiratory chain, measurement of COX-activity in BAT of knockout mice was one focus of our experiments. With increased uncoupling protein 1 (Ucp1) expression and activation during cold exposure, COX-activity increases to compensate for an increased proton leak^10^,^11^. This can principally be achieved either by an increase in mitochondrial abundance per cell, an increased abundance of assembled COX holoenzyme per mitochondrial mass, or by modifications of enzyme activity by phosphorylation^45^ or by an exchange of subunit isoforms. The latter is a COX-unique property with regard to respiratory chain complexes^46^ as can be demonstrated by deletion of single, organ-specific isozymes. For instance, deletion of Cox4i2 (lung-specific-isoform of Cox4) in the lung^47^, Cox6a2 (heart-type isoform of Cox6a) in heart and skeletal muscle^48^,^49^, and Cox7a1 in heart and skeletal muscle^21^,^22^ all affect COX-activity in the respective tissue. In heart of Cox7a1-ablated mice, Cox7a2 protein is 5-fold upregulated compared to wildtype (WT) mice^21^. This seemingly compensatory Cox7a2 expression, however, failed to rescue the reduced COX-activity caused by Cox7a1 ablation. In murine BAT, overall COX-activity is increased in response to cold-exposure, but unaffected by Cox7a1 ablation (Fig. 2). Although not observed at the transcript level (Fig. 1C), Cox7a2 protein may be able to functionally complement the loss of Cox7a1 in BAT but not in heart and skeletal muscle.

To directly investigate the effect of Cox7a1 ablation on *in vivo* BAT activity, we determined BAT-derived heat production (HP) by a norepinephrine (NE) test. This is considered the gold standard to determine maximal capacity for non-shivering thermogenesis (NST) in mice^50^. In line with published data^51^, both basal metabolic rate (BMR) and NST capacity of mice were strictly dependent on housing temperature and acclimation time, thus maximizing NST capacity after 4 weeks at 4 °C. Additionally, we observed Ucp1 expression in both BAT (Fig. 3C) and WAT (Fig. 6B) to be strongly increased by cold exposure. Since brite adipocytes were recently demonstrated to dissipate energy as heat^5^,^6^ one may speculate that alterations in NST capacity are the result of Ucp1 expression in both BAT and WAT, although the particular *in vivo* contribution of each tissue remains a topic for future studies. All tested parameters, however, were unaffected by Cox7a1 ablation, suggesting brown/brite adipocyte function to be independent of Cox7a1.

We further employed a thermal imaging approach to monitor interscapular skin surface temperature (iSST) of new-born mice obtained from heterozygous Cox7a1 breeding pairs and compared them to Ucp1-KO pups with a well characterised BAT defect^25^. Cox7a1 expression in mice is first detectable on embryonic day 17^19^ and Cox7a1 ablation may thus affect BAT activity in pups at subthermoneutral conditions (i.e., at room temperature). iSST of homozygous Ucp1 ablated mice was lower compared to wildtype and heterozygous littermates. Similar observations were recently made in adult Ucp1 knockout mice^52^, suggesting this imaging method to serve as a reliable and easy tool for the detection of defective NST. Cox7a1-KO pups, however, did not differ in iSST and thus BAT activity as compared to WT littermates. This observation is in line with results obtained from NE-tests performed with adult animals. We found no evidence for an essential role of Cox7a1 in cold-stimulated BAT function, either in adult or in new-born mice.

We conclude that Cox7a1 is dispensable for NST and ablation of Cox7a1 therefore does not require the counteracting function of other tissues. Indeed, we studied the role of Cox7a1 for BAT function in a constitutive KO model and one may speculate whether a putative impairment of NST is masked by a compensatory mechanism in another tissue. We found, however, no indication on the molecular, cellular and physiological level that may explain the requirement for alternative heat production on the organismic level. In a hypothetic scenario, where compensation of defective NST would be required, increased skeletal muscle shivering may classically be involved. Ablation of Cox7a1 in skeletal muscle diminishes maximal performance and endurance capacity during exercise challenge^22^, a phenotype that may be potentiated by cardiomyopathy^21^. Considering acute or long-term cold-exposure as exercise challenge when NST is impaired, it seems questionable whether shivering could sufficiently replace impaired NST in Cox7a1-KO mice. In fact, *in vivo* there is no need for compensation as NST capacity assessed by NE-test was normal in Cox7a1-KO mice whereas it is strongly diminished in Ucp1-KO mice^51^. Moreover, KO of Cox7a1 did not affect Ucp1 levels in WAT (Fig. 6B), indicating similar levels of WAT-derived heat production in WT and KO mice. In contrast, mouse models with impaired BAT function, like Ucp1-KO and BMP receptor-KO mice, exhibit increased expression of browning markers in WAT^51^,^53^,^54^.

Our results provide no evidence for any impairment of NST in Cox7a1-KO mice and therefore we suggest that the function of subunit 7a for COX activity in cold-activated BAT is carried out by the alternative isoform Cox7a2. In most tissues throughout the body, Cox7a2 represents the sole isoform for subunit 7a and therefore seems to be essential for COX function. Conditional knockout of either one or the other isoform may represent an appropriate strategy for future studies to elucidate the relevance of this subunit for COX activity and NST, and to particularly investigate the differential role of Cox7a1 in different tissues.

The results of our previous proteome study^9^ suggested an important role for Cox7a1 in cold-exposed mice. In the present study, the presence of Cox7a1, however, was not required to maintain body temperature via BAT-derived NST at subthermoneutral conditions. Beyond its function for adaptive NST, BAT is hypothesised to be involved in the response to hypercaloric feeding, a mechanism referred to as diet-induced thermogenesis (DIT)^30^. DIT involves BAT as a central regulator in the metabolic control of body mass development, thus dissipating excess energy derived from food via Ucp1, irrespective of any adaptive thermoregulatory requirement. The nature of such a phenotype appears to be dependent on housing temperature. This is evident in Ucp1 ablated mice fed a high fat diet (HFD), which are lean at room temperature but may become obese under thermoneutral conditions^55^. This indicates DIT to be masked under conditions when Ucp1-mediated adaptive HP is primarily required to maintain normothermia. To investigate a putative role of Cox7a1 in BAT-mediated DIT, we subjected WT and Cox7a1-KO mice to a feeding regimen known to induce obesity and impaired glucose tolerance^31^. HFD feeding at 31 °C induced increased body mass gain compared to control diet (CD) feeding, which was observable after only half a week (Fig. 7A). Total body mass gain (Fig. 7C) as well as glucose tolerance at the end of the experiment (Fig. 7D), however, was not influenced by the presence or absence of Cox7a1. We conclude that Cox7a1 does not contribute to the BAT-mediated response of HFD feeding.

An interesting incidental finding is that glucose tolerance was not altered by HFD feeding at thermoneutrality (Fig. 7E), although it usually is in C57BL/6J mice at room temperature^31^,^56^. A role for BAT in the regulation of glucose homeostasis is currently emerging in both mice^57^ and men^39^. This effect may thus be due to minimised BAT activity under thermoneutral housing. Indeed, it has been shown that the adverse effect of HFD on glucose tolerance is abrogated in rats when these are acclimated to 4 °C^58^. We speculate that the similar glucose tolerance of diet groups at thermoneutrality reflects an impaired glucose tolerance in CD-fed mice rather than an improved one in HFD-fed mice. This remains to be clarified in future studies.

In summary, Cox7a1 is a highly cold-responsive protein of BAT, a widely used marker gene for brown and brite adipocytes and essential for maximal COX-activity in heart and skeletal muscle tissue. Unexpectedly, in BAT Cox7a1 is expendable for COX enzyme activity, for cold induced tissue recruitment, and for heat production of new-born and adult mice of different temperature acclimation states. This phenotype may be caused by the alternative isoform Cox7a2, complementing the loss of Cox7a1 in BAT. Furthermore, we found no evidence for a role of Cox7a1 in WAT browning or DIT. Taken together, the brown and brite versus white adipocyte specific isoform Cox7a1 is not required to develop and maintain the key functional differences between these very different cell types.

## Methods

### Animals and housing

To establish a Cox7a1 knockout mouse line in our specific pathogen free facility we used founder mice of the Cox7a1 knockout mouseline originally described by Hüttemann and colleagues^21^. C57BL/6J wildtype (WT) and homozygous Cox7a1 knockout mice (KO) were obtained from heterozygous breeding pairs. Offspring was weaned at three weeks of age. All animals were permanently housed at a 12/12 hour light/dark cycle at room temperature (23 °C ± 1°C) with *ad libitum* access to standard rodent chow diet (V1124-300, Ssniff, Soest/Germany) and water prior to the beginning of experiments. Experimental housing at 31 °C and 4 °C was conducted in constant climate cabinets (HPP749, Memmert, Schwabach/Germany) at 65% relative humidity with *ad libitum* access to chow food and water unless stated otherwise. Tissues were dissected immediately after death of mice via CO_2_ exposure, snap-frozen in liquid nitrogen, and stored at -80 °C. All animal experimentation was conducted in accordance with the German animal welfare law. Animal experiments were performed with permission from the district government of Upper Bavaria (Regierung von Oberbayern, reference number AZ 55.2-1-54-2532-16-12).

### Genotyping

Tail tips or ear punches were taken from weaned mice and digested in 10 mM Tris (pH 8.3), 50 mM KCl, 0.45% Tween 20, 0.45% Nonidet P40 and 0.25 mg/ml proteinase K at 65 °C for at least 4 h. One μl of this solution was used as template for genotyping as described^21^ using the following PCR conditions: initial denaturation 7 min at 95 °C, 43 amplification cycles with 10 sec at 95 °C, 20 sec at 60 °C and 30 sec at 72 °C followed by 5 min final elongation at 72 °C.

### RNA isolation and quantitative real-time PCR (qRT-PCR)

Frozen tissue samples (brown adipose tissue from the suprasternal region (supraBAT), heart, liver, subcutaneous inguinal white adipose tissue (iWAT) and visceral gonadal white adipose tissue (gWAT)) were homogenised in TRIsure (Bioline, London/UK) according to the manufacturer’s instructions. Precipitated RNA was transferred on spin columns (SV Total RNA Isolation System, Promega, Madison WI/USA) and centrifuged for 15 sec at 8000 g at room temperature. The residual volume was added to the column and centrifuged for 1 min at 12000 g at room temperature. Further processing was performed according to the manufacturer’s protocol. RNA was eluted in 50 μl nuclease-free water and RNA concentration was determined spectrophotometrically (Infinite 200 PRO NanoQuant, Tecan, Männedorf/Switzerland). RNA integrity was validated using the Bioanalyzer system (RNA 6000 Nano Kit, Agilent Technologies, Santa Clara CA/USA). Reverse transcription into cDNA was performed with 500 ng RNA in a final volume of 10 μl (Quantitect Reverse Transcription Kit, Quiagen, Hilden/Germany).

qRT-PCR was conducted on 384 well plates in a total volume of 12.5 μl comprising 6.25 μl SensiMix SYBR No-ROX (Bioline, London/UK), 250 nM forward and reverse primers, and 1 μl template cDNA (LightCycler 480 System, Roche Diagnostics, Rotkreuz/Switzerland). Transcript levels of target genes were normalised to general transcription factor 2b (Gtf2b) expression (Fig. 1B). In the respective experiments, large temperature dependent variability of individual genes required normalization to the mean of the following housekeeping genes: Gtf2b, eukaryotic translation elongation factor 2 (Eef2), heat shock protein 90 alpha (cytosolic), class B member 1 (Hsp90ab1) and TATA box binding protein (Tbp) (Fig. 1C). qRT-PCR primers were produced by Eurofins MWG Operon (Ebersberg/Germany).

*Cox7a1* 5′-CCGACAATGACCTCCCAGTA-3′ and 5′-TGTTTGTCCAAGTCCTCCAA-3′

*Cox7a2* 5′-CCCTCCTCTACAGAGCCACA-3′ and 5′-CGAGCGTTGATGAAACTGAA-3′

*Eef2* 5′-ACCTGCCTGTCAATGAGTCC-3′ and 5′-CAGCATGTGGCAGTATCAGG-3′

*Gtf2b* 5′-TGGAGATTTGTCCACCATGA-3′ and 5′- GAATTGCCAAACTCATCAAAACT-3′^59^

*Hsp90ab1* 5′-AGGAGGGTCAAGGAAGTGGT-3′ and 5′-TTTTTCTTGTCTTTGCCGCT-3′

*Tbp* 5′-ACTTCACATCACAGCTCCCC-3′ and 5′-CTTCGTGCAAGAAATGCTGA-3′

*Ucp1* 5′-TCTCTGCCAGGACAGTACCC-3′ and 5′-AGAAGCCCAATGATGTTCAG-3′

### Measurement of cytochrome c oxidase (COX) activity

Frozen samples of interscapular BAT (iBAT) were homogenised in 10 mM Hepes (pH 7.4), 40  mM KCl, 2 mM EGTA, 10  mM KF, 1% Tween 20, 2 μM oligomycin and 1 mM PMSF. Homogenates were ultrasonicated and centrifuged for 2 min at 16000 g. The supernatant was collected, centrifuged a second time and cleared from residual fat. Protein concentration of the homogenate was determined spectrophotometrically at 540 nm using the Biuret-method in 80 mM NaOH, 8 mM C_4_H_4_KNaO_6_(H_2_O)_4_, 3 mM CuSO_4_(H_2_O)_5_, 4.5  mM KI and 0.1% sodium deoxycholate. COX activity was measured polarographically with a Clark-type oxygen electrode (Rank Brothers, Cambridge/UK) in a total volume of 700 μl (50 mM KH_2_PO_4_, 2 mM EGTA, 1% Tween 20, 2 μM oligomycin and 20  mM ascorbic acid) in a closed chamber at 25 °C either in the presence of 5 mM ADP or an ATP regenerating system (5 mM ATP, 5 mM MgSO_4_, 10  U/ml pyruvate kinase, and 10 mM phosphoenolpyruvic acid). Oxygen consumption was recorded during a titration of cytochrome *c* (1–30  μM).

### SDS-PAGE and Western Blot

SDS-PAGE was conducted with total protein from iBAT homogenates (see above). For WAT analysis, iWAT was homogenised in 5 μl/mg 50 mM Tris, 1% NP-40, 0.25% sodium deoxycholate, 150 mM NaCl, 1  mM EDTA and 0.1% protease-/phosphatase inhibitor cocktail (Sigma, St. Louis MO/USA). Homogenates were centrifuged for 15 min at 16000 g. The supernatant was centrifuged a second time for 2 min at 16000 g and cleared from residual fat. Protein concentration was determined with the bicinchoninic acid (BCA) method (Pierce^TM^ BCA Protein Assay Kit, Thermo Scientific, Waltham MA/USA) according to the manufacturer’s instructions. Thirty μg of iBAT and iWAT protein were resolved in a 12.5% SDS-PAGE and transferred to a nitrocellulose membrane. Primary antibodies were applied to detect uncoupling protein 1 (Ucp1, rabbit anti-hamster IgG known to reliably detect mouse UCP1) and pan-actin (mouse IgG, Merck Millipore, Billerica MA/USA). Primary antibodies were detected using IR-dye conjugated secondary antibodies (IRDye 800CW and 680CW, LI-COR, Lincoln NE/USA). IR signal was detected using a LI-COR Odyssey imager. Image analysis was performed using the LI-COR Odyssey Software version 3.0. Additional information on SDS-PAGE and Western Blot is provided as Supplementary Material.

### Histology

One lobe of every iWAT depot was placed in 4% paraformaldehyde and 0.0024% picric acid immediately after dissection and fixed for several days, followed by dehydration (TP1020, Leica, Wetzlar/Germany) and paraffin-embedding (EG1150C, Leica). Five μM sections (RM2255, Leica) were mounted on object slides and dried for at least 24 h at 37 °C. Hematoxylin-eosin (HE) staining was performed using the Leica ST5020 multistainer. Stained sections were covered with mounting medium (Carl Roth, Karlsruhe/Germany). Sections were analysed under a microscope (DMI4000B, Leica) using similar adjustments for all slides.

### Indirect calorimetry and norepinephrine (NE) test

Male and female WT and KO mice were subjected to two consecutive NE-tests to determine maximal norepinephrine-stimulated heat production (HP). All animals were single-caged one week prior to the first test, which was performed at the age of 12-13 weeks in a room temperature-acclimated state. The second test was performed four weeks later after acute (4 days) or chronic (28 days) cold-exposure (4 °C). Rectal body temperature (Almemo 2490, Ahlborn, Holzkirchen/Germany) was measured once a week and 4 days prior to the second test between 7:30 and 9:00 am.

In the morning of the test day, mice were placed in metabolic cages (3L volume) without food and water, transferred to a climate cabinet (TPK 600, Feutron, Greiz/Germany) preconditioned to 31 °C and connected to the indirect calorimetry setup (Phenomaster, TSE Systems, Bad Homburg/Germany) to measure basal metabolic rate (BMR) during fasting (8:00 am–12:00 pm). The air from the cages was extracted over a period of 1 min every 7 min with a flow rate of 33 l/h, dried in a cooling trap and analysed for O_2_ and CO_2_ content. O_2_ consumption and CO_2_ production [ml/h] were calculated via comparison of the air from the cages with the air from an empty reference cage. BMR [ml O_2_/h] was calculated as the lowest mean of three consecutive oxygen consumption values, which had a coefficient of variation less than 5%.

After BMR measurements, the cages were removed from the climate cabinet, which was subsequently altered over about 20 min to 26 °C (first NE-test) or 20 °C (second NE-test), respectively, to avoid NE-stimulated hyperthermia of cold-acclimated mice. Mice were injected subcutaneously with 1  mg/kg NE. O_2_ consumption and CO_2_ production was recorded for 60–75 min with a 1 min read every 3 min. O_2_ consumption data were smoothed by calculating the mean of three consecutive values, and maximal NE-stimulated (NE_max_) O_2_-consumption was obtained from the highest individual mean. Metabolic rates (BMR and NE_max_) were converted into HP [mW] as previously described^51^. Capacity for non-shivering thermogenesis was calculated as the difference between maximal (NE_max_) and minimal (BMR) HP. All animals were killed and dissected as described above within 1 h after the end of the second NE-test.

### Thermal imaging

Thermal imaging was performed with new-born mice obtained from heterozygous breeding of Ucp1 (C57BL/6J background) and Cox7a1 knockout mouse lines within the first three days of life. Pups were placed in 6-well cell culture plates and an individual series of pictures (at least 6) was taken of each litter (T890 thermal imager, Testo, Lenzkirch/Germany). Mice were killed by decapitation and post mortem genotyping was performed from tail tips. Images were analysed using the IRSoft Software (version 3.1, Testo, Lenzkirch/Germany), detecting the warmest spot within a defined region in the upper dorsal area, thus obtaining one data point on each picture for each animal, referred to as interscapular skin surface temperature (iSST). A mean iSST-value was calculated for each animal using data from serial pictures as technical replicates. To standardise data from different litters, an average iSST was calculated for heterozygous animals (mean_het_) within each litter, and data are expressed as difference between iSST mean_het_ and iSST of each individual animal within this litter.

### High fat diet (HFD) feeding and oral glucose tolerance tests

At 8 weeks of age, male WT and KO mice were placed in climate cabinets at 31 °C (2–3 mice per cage) and fed with a control diet (CD, S5745-E702, Ssniff, Soest/Germany, 11% energy from fat). After 2 weeks, half of the mice remained on control diet, while the other half was switched to a HFD (S5745-E712, Ssniff, Soest/Germany, 45% energy from fat) for 4 weeks. Body mass development was monitored two times per week throughout the experiment. Body composition was measured at the beginning and at the end of HFD-feeding with a nuclear magnetic resonance instrument (mq 7.5 NMR analyser, Bruker, Billerica MA/USA). At the end of the experiment oral glucose tolerance tests (oGTT) were performed. Mice were single caged without food in the morning of the test day and fasted for 6 h (8:00 am–2:00 pm). Mice received an oral glucose load of 2.8  g/kg lean mass. Blood was taken from incised tail tips and blood glucose concentration was measured before (0 min) and 15, 30, 60, and 120 min after gavage with a whole blood monitor (FreeStyle Lite, Abbott, Wiesbaden/Germany). Fasting and oGTT procedures were performed at room temperature. Total area under the curve (AUC) of blood glucose was calculated by the trapezoidal method^60^.

### Statistics

All data are presented as means ± standard deviation (SD) or as single values of individual animals with the group mean indicated as horizontal line. Statistical analysis was performed using GraphPad Prism 6 (GraphPad Software Inc., La Jolla CA/USA) and SigmaPlot 12.0 (Systat Software Inc., San Jose CA/USA). P-values < 0.05 were considered statistically significant. Asterisks indicate significance. Statistical tests were applied as indicated in figure legends.

## Additional Information

**How to cite this article**: Maurer, S. F. *et al.* The brown and brite adipocyte marker Cox7a1 is not required for non-shivering thermogenesis in mice. *Sci. Rep.* **5**, 17704; doi: 10.1038/srep17704 (2015).

## Supplementary Material

Supplementary Materials
