# Supplementary material for: The brown and brite adipocyte marker Cox7a1 is not required for non-shivering thermogenesis in mice
Source: Sci Rep. 2015 Dec 4;5:17704. doi: 10.1038/srep17704 (PMC4669493; doi:10.1038/srep17704)

**The brown and brite adipocyte marker Cox7a1 is not required for non-shivering thermogenesis in mice.**

Stefanie F. Maurer^1^, Tobias Fromme^1^, Lawrence I. Grossman^2^, Maik Hüttemann^2^ and Martin Klingenspor^1,*^

^1^Chair of Molecular Nutritional Medicine, Technische Universität München, Else Kröner-Fresenius Center for Nutritional Medicine & ZIEL - Institute for Food and Health, 85350 Freising‑Weihenstephan, Germany

^2^Center for Molecular Medicine and Genetics, Wayne State University School of Medicine, Detroit, MI 48201, USA

*corresponding author (contact: mk@tum.de)

**SUPPLEMENTARY METHODS**

SDS-PAGE was conducted with total protein derived from iBAT and iWAT tissue homogenates. Target proteins were identified by application of a molecular weight marker (PageRuler™ Prestained Protein Ladder, 10 to 180 kDa, Thermo Scientific, St. Louis, MO/USA).

For iBAT analysis (Supplementary Figure 1), primary antibodies were co-incubated for 1.5 hours to detect pan‑actin (42 kD, host mouse, Anti-Actin clone c4, Merck Millipore, Billerica MA/USA), uncoupling protein 1 (Ucp1, 32 kD, host rabbit, custom-made) and cytochrome *c* oxidase subunit 4 (Cox4, host rabbit, Cell Signalling Technology, Danvers MA/USA; 17 kD according to manufacturer’s information, but actually detected at approx. 14 kD with mouse adipose tissue). IR-dye conjugated secondary antibodies (goat-anti-rabbit 800CW, donkey-anti-mouse 680CW; LI-COR, Lincoln NE/USA) were co-incubated for 1.5 hours.

For iWAT analysis (Supplementary Figure 2), the nitrocellulose membrane was horizontally cleaved between 35 and 40 kD (see dashed line in Supplementary Figure 2) after protein transfer. The upper part was co‑incubated for 1.5 hours with primary antibodies to detect heat shock protein 60 (Hsp60, 60 kD, host goat, Santa Cruz Biotechnology, Dallas TX/USA) and pan-actin. The lower part was co‑incubated with primary antibodies targeting Ucp1 and Cox4. Secondary antibodies were incubated for 1.5 hours (upper part: donkey-anti-goat 800CW and donkey-anti-mouse 680CW co-incubation; lower part: goat‑anti-rabbit 800CW).

**SUPPLEMENTARY FIGURE LEGENDS**

**Supplementary Figure 1:** Original image of Western Blot analysis in iBAT generated with the Odyssey imaging system. Mice were housed at 31°C for 2 weeks, at 23°C and at 4°C for 4 days or 8 days, respectively. Samples derived from differentially treated mice were loaded on the same SDS-PAGE and are denominated as ‘other’. WT=wildtype, KO=Cox7a1-knockout, M=molecular weight marker. Red and green signals are derived from secondary antibodies conjugated with IR-dye 680CW and 800CW, respectively. Size distribution of the molecular weight marker and associated allocation of target proteins are indicated.

**Supplementary Figure 2:** Original image of Western Blot analysis in iWAT generated with the Odyssey imaging system. Male and female mice were housed at 23°C and at 4°C for 4 days or 28 days, respectively. iBAT was used as positive control for Ucp1 detection. Dashed horizontal line indicates cleavage site of membrane after blotting.

**Supplementary Figure 1**


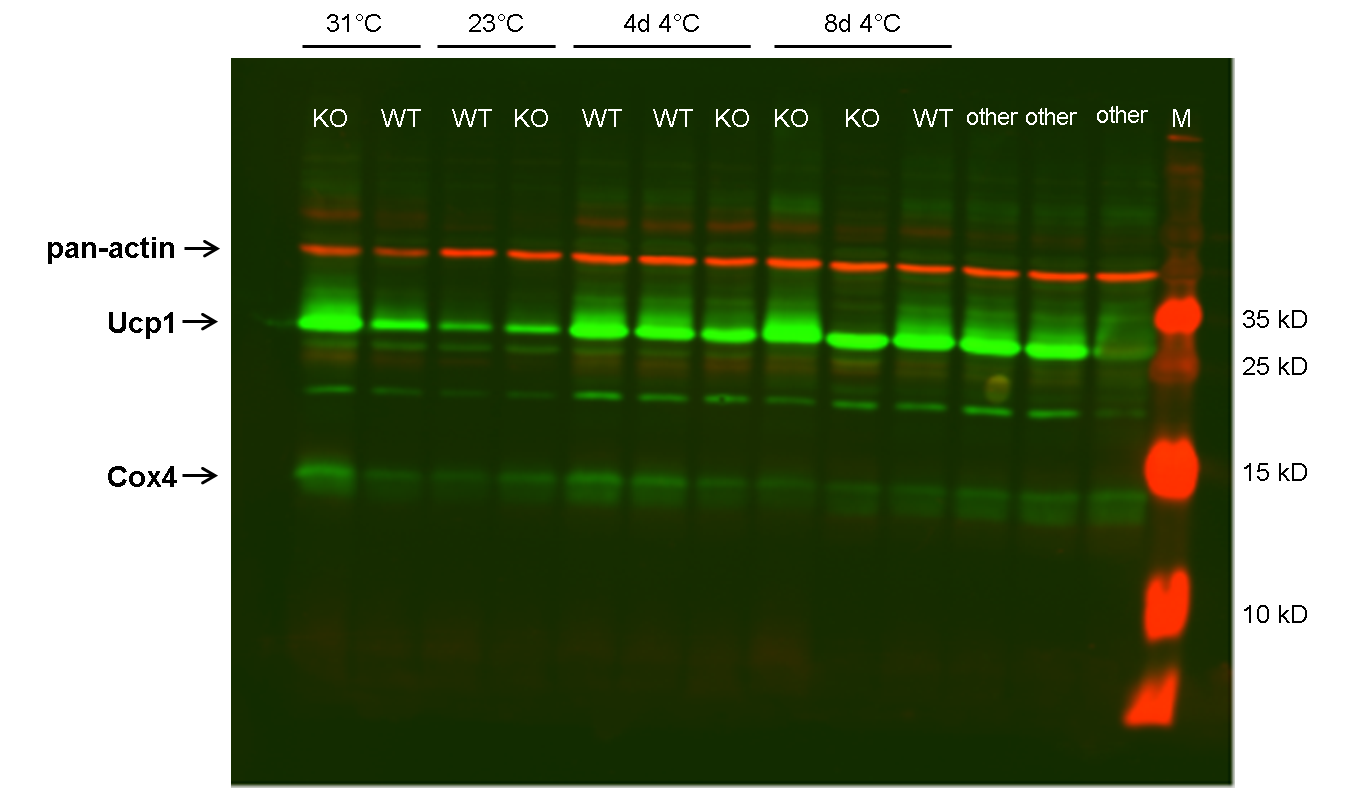


**Supplementary Figure 2**


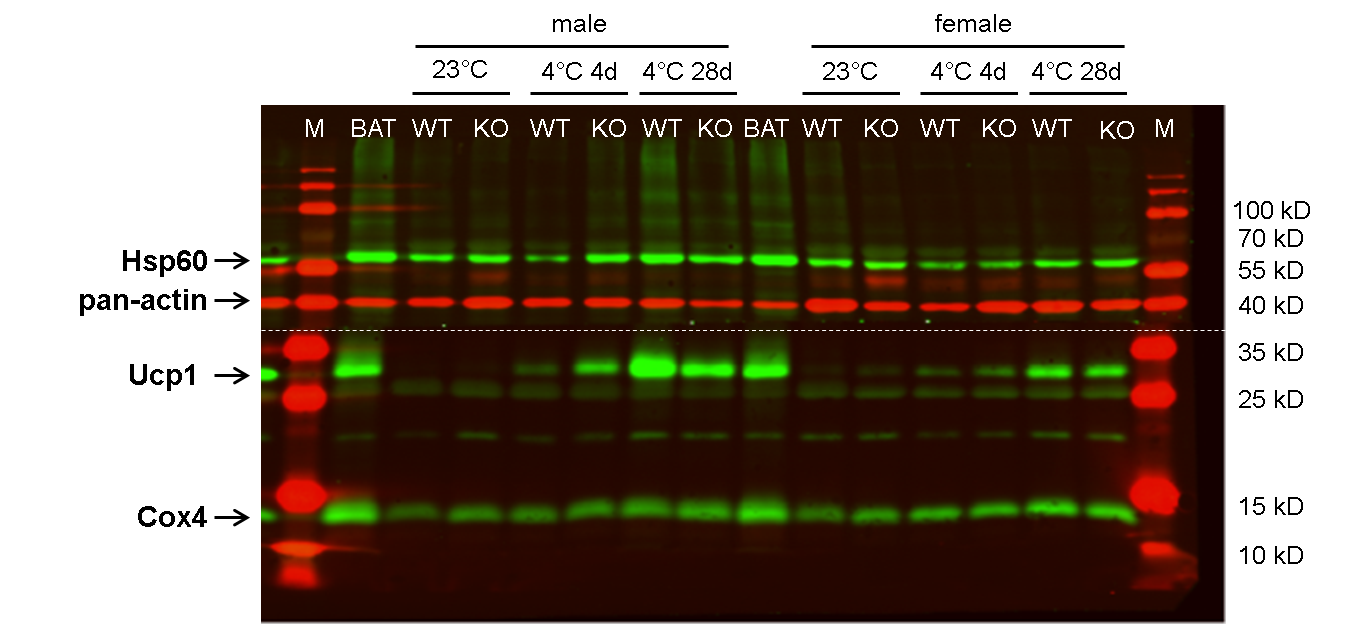

Supplement: Supplementary Materials [file srep17704-s1.doc]
